# Nephrolithiasis and nutrition in obesity

**DOI:** 10.1186/1753-6561-6-S3-P45

**Published:** 2012-06-01

**Authors:** Laura Soldati, Elena Dogliotti, Annalisa Terranegra, Tiziana Meschi, Antonio Nouvenne, Beatrice Prati, Loris Borghi, Alessandro Leone, Simona Bertoli, Giuseppe Vezzoli, Alberto Battezzati

**Affiliations:** 1 Dep. Medicine, Surgery, Dentistry, San Paolo Hospital, Milan, 20100, Italy; 2 Nephrology and Dialysis Unit, San Raffaele Hospital, Milan, 20100 Italy; 3 Dep. Clinical Sciences, University of Parma, Parma, 43121, Italy; 4 Dep. Food Science, Technology and Microbiology, International Center for the Assessment for Nutritional Status, University of Milan, Milan, 20100, Italy

## Background

Obesity is a risk factor for nephrolithiasis (NL). According to an American study of a large cohort with a BMI >30, males increased the risk of NL of 30% and females of 200%. Moreover, diet plays an important role as NL risk factor, mainly in those western countries characterized by a large meet consume. This epidemiology study investigated the NL frequency in an obese Italian population, also considering the relation with metabolic syndrome and interaction with diet.

## Materials and methods

Nutritional assessment included: nutritional questionnaires, anthropometry (BMI and waist circumference, body composition estimated by impedance and biochemistry (fasting glucose, serum lipids and transaminases). The presence of nephrolithiasis, osteoporosis, arterial hypertension, diabetes mellitus and metabolic syndrome were noted. Statistical analysis was performed by the SPSS software.

## Results

We studied 532 obese Caucasian subjects (M/F 144/388; age 46±13.1; BMI 29.7±5.85 kg/m^2^). The stone formers (SF) were 41 (7.7% of the analyzed population, M 9,7%, F 6,9%; age 54.2±11.7 yrs; BMI 30.6±5.8 kg/m^2^). Non-stone formers (NSF) were 491 (92.3%, M/F 130/361; age 45.3±12.9 yrs [p=0.001]; BMI 29.6±5.8 kg/m^2^). The percentage of subjects with osteoporosis was higher in SF than in the NSF (15.79% vs 5.84%, p=0.018). In comparison with NSF, SF had higher mean waist size (101.5 vs 96.6 cm, p=0.02), systolic pressure (SF vs NSF, 130.5±15.3 vs 124.4±13.5 mmHg, p=0.007) and higher fasting serum glucose concentration (104±27.4 vs 94±15.3 mg/dL, p=0.02). Diet analysis did not show differences between SF and NSF, except for a higher intake of butter, wine and white meet in SF.

## Conclusions

Obese SF had an increased risk of osteoporosis, hypertension, diabetes and metabolic syndrome than obese NSF. This cohort of obese subjects presented a slight increase of prevalence in NL than in general Italian population that is around 5%. The prevalence of stone formers in this Italian obese population is lower than that observed in American obese subjects. This difference could be due to dissimilar genetic background or dietary habits. These findings suggest that Mediterranean diet may have a protective role against NL.

